# Editorial: Integrating Whole Genome Sequencing Into Source Attribution and Risk Assessment of Foodborne Bacterial Pathogens

**DOI:** 10.3389/fmicb.2021.795098

**Published:** 2021-11-26

**Authors:** Frederique Pasquali, Daniel Remondini, Emma Louise Snary, Tine Hald, Laurent Guillier

**Affiliations:** ^1^Department of Agricultural and Food Sciences, Alma Mater Studiorum - University of Bologna, Bologna, Italy; ^2^Department of Physics and Astronomy, Alma Mater Studiorum - University of Bologna, Bologna, Italy; ^3^Department of Epidemiological Sciences, Animal and Plant Health Agency (APHA), Addlestone, United Kingdom; ^4^National Food Institute, Technical University of Denmark, Kongens Lyngby, Denmark; ^5^Department of Risk Assessment, Agence Nationale de Sécurité Sanitaire de l'Alimentation, de l'Environnement et du Travail (ANSES), Maisons-Alfort, France

**Keywords:** source attribution, microbial risk assessment, whole genome sequencing, metagenomics, foodborne pathogens

Source attribution and microbial risk assessment have proved to be crucial to identify and prioritize food safety interventions as to effectively control the burden of human illnesses (Cassini et al., 2016; Mughini-Gras et al., 2018a, 2019). By comparing human cases and pathogen occurrences in selected animal, food, and environmental sources, microbial subtyping approaches were successfully applied to pinpoint the most important sources of *Salmonella, Campylobacter*, Shiga toxin-producing *Escherichia coli*, and *Listeria monocytogenes* (Hald et al., 2004; Mullner et al., 2009a,b; Barco et al., 2013; Nielsen et al., 2017; Mughini-Gras et al., 2018b; Cody et al., 2019). Microbial risk assessment has been applied to assess known or potential adverse health effects resulting from human exposure to food-borne hazards. Through a scientific structured approach (FAO and WHO, 2021), microbial risk assessment helps to identify and quantify the risk represented by specific foods and the critical points in these foods' production chains for microbial control (Cassini et al., 2016; FAO and WHO, 2021). For both source attribution and risk assessment, one key challenge has been to define the hazard in question: is the whole foodborne pathogen species a hazard, or only some of its subtypes? In this regard the choice of the subtyping method becomes crucial. In recent years, Whole Genome Sequencing (WGS) has represented a major benefit for more targeted approaches, no longer focused on the species/genus level but at the level of subtypes (Franz et al., 2016; Fritsch et al., 2018; EFSA Panel on Biological Hazards, 2019). Besides WGS, metagenomics showed potentialities in source attribution. In particular, this approach was useful in attributing the source of environmental contamination by comparing the abundances of source-specific genetic markers (i.e., resistome) in different reservoirs (Gupta et al., 2019).

Therefore, this special issue focuses on traditional and novel source attribution approaches applied on molecular, WGS, and metagenomic data as well as on a fine-tuning genetic characterization of foodborne pathogens useful for hazard identification and characterization. In particular, one study compares the outputs of a modified Hald model, which was applied to different subtyping input data of *S*. *enterica* Typhimurium and its monophasic variant (Arnold et al.) whereas two studies proposed a novel network approach and a method based on the core-genome genetic distance to attribute human infections of *S. enterica* Typhimurium monophasic variant and *S*. *enterica* Derby using WGS as input data (Merlotti et al.; Sévellec et al.). Another study by Duarte et al. included the relative abundance of antimicrobial resistance (AMR) associated genes (resistome) as metagenomic input data in an AMR source attribution study. Finally, two studies were focused on the molecular and genomic characterization of human isolates of *Campylobacter jejuni* and *C. coli* from China and of *Listeria monocytogenes* isolates collected from ready-to-eat meat and processing environment from Poland (Zhang et al.; Kurpas et al.).

Arnold et al. performed a source attribution study including the genomes of *S*. *enterica* Typhimurium and its monophasic variant of 596 human sources and 327 animal sources from England and Wales between 2014 and 2016. Data from Seven Loci Multi Locus Sequence Typing (7-loci MLST), core-genome MLST (cg-MLST), and SNP calling were compared as input data. By applying a modified Hald model, 60% of human genomes were attributed to pork. Comparing different input data, results highlighted MLST as the method with the lowest fit and the lowest discriminatory power.

Merlotti et al. applied a network approach to 351 human and animal genomes of *S*. *enterica* Typhimurium and its monophasic variant collected from 2013 to 2014. Three datasets of whole-genome MLST (wgMLST), cgMLST, and SNPs were used as input data. Genomes were clustered based on their genetic similarities. Interestingly, a higher percentage of cluster coherence was reported for animal sources in comparison to country and year of isolation, suggesting animal sources as the major driver of cluster formation. The approach showed to be effective in attributing up to 97.2% of human genomes to animal sources represented in the dataset. Among these genomes, the majority (84%) was attributed to pigs/pork. No significant differences were highlighted by comparing the three different input datasets.

Core genome analysis was the approach applied by Sévellec et al. to attribute human sporadic cases of *S*. *enterica* Derby that occurred in France in 2014–2015 to non-human reservoirs. The authors analyzed 299 *S*. *enterica* Derby genomes corresponding to all *S. enterica* Derby sporadic human cases registered in the time frame, along with 141 non-human genomes. Within the non-human genomes, three main genomic lineages were detected in France: ST39-ST40 and ST682 associated to pork and ST71 associated to poultry. Within human genomes, 94% of *S*. *enterica* Derby clustered within the three genetic groups associated with pork, identifying this animal reservoir as the major contributor of *S*. *enterica* Derby to sporadic human cases in France.

Relative abundance of antimicrobial resistance genes in shotgun metagenomic data was chosen in an antimicrobial resistance source attribution study by Duarte et al.. Starting from the assumption that fecal resistomes are source related, authors compared the resistomes of pooled fecal samples of pigs, broilers, turkeys, and veal calves with the resistomes of individual fecal samples of humans occupationally exposed to livestock production. Five supervised random forest models were applied on a total of 479 observations. Among the four livestock species, the results indicated that pigs have the resistome composition closest to the composition of the human resistome suggesting that occupational exposure to AMR determinants was higher among workers exposed to pigs than workers of broiler farms.

Zhang et al. characterized genetic diversity and antimicrobial resistance of 236 *Campylobacter jejuni* and *C. coli* isolates collected from 2,945 individual stool samples of hospitalized patients with diarrhea in Beijing from 2017 to 2018. MLST results confirmed the high genetic diversity among isolates as well as CC21 as the most common clonal complex of *C. jejuni* in diarrhea patients in China. Clonal complex CC828 was the most frequently identified among *C. coli* isolates. Regarding antimicrobial resistance, rates higher than 88% were identified for the antimicrobials nalidixic acid, ciprofloxacin, and tetracycline.

Last but not least, Kurpas et al. genetically characterized 48 *L. monocytogenes* isolates of PCR-serogroup IIb and IVb collected from ready-to-eat food and food processing environments. Additionally, the authors compared them with public genomes collected from humans in Poland. Among food isolates, 65% belonged to CC1, CC2, and CC6 already described as hypervirulent strains in humans. The clonal complex CC5 was also identified; mostly collected from food processing environments and belonging to PCR-serogroup IIB. Genomes of this clonal complex showed mutations in the *inlA* gene and a deletion of 144 bp in the *inlB* gene suggesting them as hypovirulent.

Based on these studies, we conclude that the application of NGS data, in particular source attribution models, shows great potential. The results are improved by becoming more specific and to the point, which is considered very valuable for the decision support process. Integrations with phenotypic tests will continue to be essential for confirmation of NGS predicted outcomes.

## Author Contributions

All authors were topic editors. FP wrote the first draft of the manuscript. DR, ES, TH, and LG wrote sections of the manuscript. All authors contributed to manuscript revision, read, and approved the submitted version.

## Conflict of Interest

The authors declare that the research was conducted in the absence of any commercial or financial relationships that could be construed as a potential conflict of interest.

## Publisher's Note

All claims expressed in this article are solely those of the authors and do not necessarily represent those of their affiliated organizations, or those of the publisher, the editors and the reviewers. Any product that may be evaluated in this article, or claim that may be made by its manufacturer, is not guaranteed or endorsed by the publisher.

## References

[B1] Barco L. Barrucci F. Olsen J. E. Ricci A. (2013). *Salmonella* source attribution based on microbial subtyping. Int. J. Food Microbiol. 163, 193–203. 10.1016/j.ijfoodmicro.2013.03.005 23562696

[B2] Cassini A. Hathaway S. Havelaar A. Koopmans M. Koutsoumanis K. Messens W.. (2016). Special issue: microbiological risk assessment. EFSA J. 14:s0507. 10.2903/j.efsa.2016.s0507

[B3] Cody A. J. Maiden M. C. Strachan N. J. McCarthy N. D. (2019). A systematic review of source attribution of human campylobacteriosis using multilocus sequence typing. Euro Surveill. 24:1800696. 10.2807/1560-7917.ES.2019.24.43.1800696 31662159 PMC6820127

[B4] EFSA Panel on Biological Hazards Koutsoumanis K. Allende A. Alvarez-Ordonez A. Bolton D. Bover-Cid S.. (2019). Scientific opinion on the whole genome sequencing and metagenomics for outbreak investigation, source attribution and risk assessment of food-borne microorganisms. EFSA J. 17:5898. 10.2903/j.efsa.2019.5898 32626197 PMC7008917

[B5] FAO and WHO (2021). Microbiological risk assessment - Guidance for food. Rome: Guidance. Microbiological Risk Assessment Series No. 36.

[B6] Franz E. Gras L. M. Dallman T. (2016). Significance of whole genome sequencing for surveillance, source attribution and microbial risk assessment of foodborne pathogens. Curr. Opin. Food Sci. 8, 74–79. 10.1016/j.cofs.2016.04.004

[B7] Fritsch L. Guillier L. Augustin J. C. (2018). Next generation quantitative microbiological risk assessment: refinement of the cold smoked salmon-related listeriosis risk model by integrating genomic data. Microb. Risk Anal. 10, 20–27. 10.1016/j.mran.2018.06.003

[B8] Gupta S. Arango-Argoty G. Zhang L. Pruden A. Vikesland P. (2019). Identification of discriminatory antibiotic resistance genes among environmental resistomes using extremely randomized tree algorithm. Microbiome 7:123. 10.1186/s40168-019-0735-1 31466530 PMC6716844

[B9] Hald T. Vose D. Wegener H. C. Koupeev T. (2004). A Bayesian approach to quantify the contribution of animal-food sources to human salmonellosis. Risk Anal. 24, 255–269. 10.1111/j.0272-4332.2004.00427.x 15028016

[B10] Mughini-Gras L. Kooh P. Augustin J. C. David J. Fravalo P. Guillier L.. (2018a). Source attribution of foodborne diseases: potentialities, hurdles, and future expectations. Front. Microbiol. 9:1983. 10.3389/fmicb.2018.01983 30233509 PMC6129602

[B11] Mughini-Gras L. van Pelt W. van der Voort M. Heck M. Friesema I. Franz E. (2018b). Attribution of human infections with Shiga toxin-producing *Escherichia coli* (STEC) to livestock sources and identification of source-specific risk factors, the Netherlands (2010-2014). Zoonoses Public Health 65, e8–e22. 10.1111/zph.12403 28921940

[B12] Mughini-Gras L. Kooh P. Fravalo P. Augustin J. C. Guillier L. David J. (2019). Critical orientation in the jungle of currently available methods and types of data for source attribution of foodborne diseases. Front. Microbiol. 10:2578. 10.3389/fmicb.2019.02578 31798549 PMC6861836

[B13] Mullner P. Jones G. Noble A. D. Spencer S. E. F. Hathaway S. French N. P. (2009b). Source attribution of food-borne zoonoses in New Zealand: a modified Hald model. Risk Anal. 7, 970–284. 10.1111/j.1539-6924.2009.01224.x 19486473

[B14] Mullner P. Spencer S. E. F. Wilson D. Jones G. Noble A. D. Midwinter A. C.. (2009a). Assigning the source of human campylobacteriosis in New Zealand: a comparative genetic and epidemiological approach. Inf. Genet. Evol. 9, 1311–1319. 10.1016/j.meegid.2009.09.003 19778636

[B15] Nielsen E. M. Björkman J. T. Kiil K. Grant K. Dallman T. Painset A.. (2017). Closing gaps for performing a risk assessment on listeria monocytogenes in ready-to-eat (RTE) foods: activity 3, the comparison of isolates from different compartments along the food chain, and from humans using whole genome sequencing (WGS) analysis. EFSA Support. Public. 14:1151E. 10.2903/sp.efsa.2017.EN-115

